# Antimycin A-Induced Mitochondrial Damage Causes Human RPE Cell Death despite Activation of Autophagy

**DOI:** 10.1155/2019/1583656

**Published:** 2019-03-17

**Authors:** Maria Hytti, Eveliina Korhonen, Juha M. T. Hyttinen, Heidi Roehrich, Kai Kaarniranta, Deborah A. Ferrington, Anu Kauppinen

**Affiliations:** ^1^School of Pharmacy, University of Eastern Finland, Kuopio, Finland; ^2^Department of Ophthalmology, Institute of Clinical Medicine, University of Eastern Finland, Kuopio, Finland; ^3^Histology Core for Vision Research, University of Minnesota, Minneapolis, MN, USA; ^4^Department of Ophthalmology, Kuopio University Hospital, Kuopio, Finland; ^5^Department of Ophthalmology and Visual Neurosciences, University of Minnesota, Minneapolis, MN, USA

## Abstract

Mitochondrial dysfunction has been implicated in a wide variety of degenerative diseases, including age-related macular degeneration. Damage to mitochondria and mitochondrial DNA accumulates with age in the postmitotic retinal pigment epithelium (RPE), which could lead to RPE cell death and trigger disease. One possible mechanism for cells to avoid cell death is mitophagy, the targeted clearance of damaged mitochondria by autophagy. Here, we induced mitochondrial damage in human RPE cells (ARPE-19 and hRPE), using antimycin A, an inhibitor of complex III of the electron transport chain, and investigated cellular viability, mitochondrial structure and function, and autophagy activity. We observed that antimycin A evoked dose-dependent cell death, a rapid loss in mitochondrial membrane potential, and a collapse of oxidative phosphorylation. Mitochondria appeared swollen and there was clear damage to their cristae structure. At the same time, cells were undergoing active autophagy and were sensitive to autophagy inhibition by bafilomycin A1 or chloroquine. These results indicate that mitochondrial dysfunction can cause significant RPE damage and that autophagy is an important survival mechanism for cells suffering from mitochondrial damage.

## 1. Introduction

Mitochondria are vital cell organelles that not only produce the majority of cellular ATP but also control cellular calcium homeostasis and regulate apoptotic pathways, among many other key functions [1]. They are also the primary source of intracellular reactive oxygen species (ROS) [2]. During normal cellular metabolism, ROS can function as important secondary messengers and there is a balance between ROS production and their detoxification by cellular antioxidant systems [2, 3]. However, dysfunctional mitochondria, marked by reduced ATP production and an increased generation of ROS, disturb this balance and have been speculated to contribute to ageing and the development of age-related diseases [1, 3, 4]. In a vicious cycle, aberrant mitochondrial ROS cause further damage to mitochondrial DNA (mtDNA), membrane lipids, and proteins, increasing mitochondrial damage and further augmenting ROS leakage. ROS generation and mtDNA damage have been found to increase with age, while there is a corresponding decline in mitochondrial function and ATP generation [4].

mtDNA is a 16569 bp loop of super-coiled, double-stranded DNA, encoding 37 genes that translate 22 tRNAs, 2 ribosomal RNAs, and 13 proteins [1, 5]. All of the proteins encoded by the mtDNA are components of the electron transport chain (ETC) and vital for cellular energy production by oxidative phosphorylation (OXPHOS). Due to a lack of protective histones and its close proximity to the ROS produced by the ETC, mtDNA is susceptible to mutations; it has been estimated to have a mutation rate 10 times more than that of nuclear DNA [5, 6]. Furthermore, the relative lack of noncoding regions and an absence of introns in mtDNA [5] mean that mtDNA mutations almost invariably cause dysfunction in ETC protein expression and, consequently, lead to a loss of mitochondrial function, i.e., energy generation declines while ROS production increases. Heteroplasmy prevents immediate consequences of mtDNA damage to cells, but as the number of mutated mtDNA molecules and ROS production increase with age, cells are at an increased risk of dying [1]. Postmitotic tissues, such as the brain, muscle, and retinal pigment epithelium (RPE), are especially vulnerable to the accumulation of mtDNA damage, as the mitochondrial genome replicates independently of the cell cycle, allowing the clonal expansion of mutated mtDNA [1, 5]. Consequently, mitochondrial dysfunction has been linked to many age-related neurodegenerative diseases, such as Parkinson's disease [7, 8] and Alzheimer's [9, 10].

There is evidence that dysfunctional mitochondria are a key factor also in the development of age-related macular degeneration (AMD), the leading cause of blindness among the elderly [11, 12]. Mitochondrial number and size, as well as the mitochondrial matrix density, are reduced in the RPE of AMD patients [13]. Mitochondrial DNA damage was found to be elevated in the retina and RPE layer of AMD patients when compared to healthy controls [14, 15] whereas the protein expression levels of several subunits of the ATP synthase as well as cytochrome c oxidase were reduced in AMD patients suffering from advanced AMD [16].

Cells can employ a specific form of macroautophagy, called mitophagy, to remove damaged mitochondria. Excessive production of the superoxide anion by the ETC is a known trigger for the induction of autophagy [17, 18], and dysfunction of the ETC and a loss of mitochondrial membrane potential (MMP) are known activators of mitophagy [18, 19]. In RPE cells, Lee et al. have shown that inhibition of complex I of the electron transport chain by exposure to rotenone triggers mitotic catastrophe and makes cells more susceptible to death by inhibition of autophagy [20]. They postulated that mitophagy was a fundamental survival mechanism for RPE cells suffering from mitochondrial damage. Overall, there is growing evidence that dysfunctional mitophagy plays an important role in AMD [21].

Here, we used antimycin A (Aa) to induce mitochondrial damage with the aim of creating a model of mitochondrial dysfunction in RPE cells and to study its consequences on cell viability and autophagy. Like complex I, complex III is a major site of ROS leakage from the ETC and its inhibition has been shown to cause mitochondrial DNA damage and apoptosis in Hep3B cells [22] and endothelial cells [23]. We analyzed the effects of Aa treatment on mitochondrial physiology and function, cellular viability, and autophagy flux. To verify the relevance of our findings, we have confirmed the data from the immortalized cell line ARPE-19 by repeating major findings in primary human RPE (hRPE) cells generated from aged donors, aged 61 to 88.

## 2. Materials and Methods

### 2.1. Cell Culture

ARPE-19 cells were obtained from the American Type Culture Collection (ATCC). Passage numbers 25 - 36 were used in all experiments. Routinely, cells were kept in DMEM/F-12 (1 : 1; Life Technologies, Carlsbad, CA, USA) supplemented with 100 U/ml penicillin (Life Technologies, Carlsbad, CA, USA), 100 *μ*g/ml streptomycin (Life Technologies, Carlsbad, CA, USA), 10% fetal bovine serum (FBS) (Thermo Fisher Scientific, Waltham, MA, USA), and 2 mM l-glutamine (Life Technologies, Carlsbad, CA, USA). The cells were passaged every 3-4 days. Primary human RPE (hRPE) cells were isolated from human cadaver eyes obtained from the Lions Gift of Sight (formerly the Minnesota Lions Eye Bank, St. Paul, MN). In accordance with the Declaration of Helsinki, eyes were obtained with the written consent of the donor or donor's family for use in medical research. Donor tissue is exempt from the process of Institutional Review Board approval. Patient data from Eye Bank records is summarized in Table 1. hRPE cells were harvested and cultured as previously described [24]. Cells in passage 3 or 4 were used. All cells were kept in an incubator, providing a humidified atmosphere with 5% CO_2_ and a stable temperature of +37°C.

### 2.2. Chemicals and Cell Treatments

In the experiments, cells were seeded into 96-well plates (toxicity studies, mitochondrial membrane potential assessments), 12-well plates (Western blot analyses, toxicity studies, transmission electron microscopy samples, and caspase 3 activity measurements), or 8-well chamber slides (transfection studies, TUNEL staining, and confocal microscopy). Cell density was 15000 cells/well in 96-well plates, 20000 cells/well in 8-well chamber slides, and 200000 cells/well on 12-well plates. ARPE-19 cells were cultured in tissue culture-treated plates, while hRPE cells were plated on well surfaces coated with Matrigel (Corning, Corning, NY, USA). The cells were incubated for 72-96 hours until fully confluent, unless otherwise stated, and were treated with varying concentrations of antimycin A (Aa) (Sigma-Aldrich, St. Louis, MO, USA), with or without pretreatment with 30 *μ*M chloroquine (CQ) (Sigma-Aldrich, St. Louis, MO, USA) or 50 nM bafilomycin A1 (BafA) (Sigma-Aldrich, St. Louis, MO, USA) in serum-free culture medium. Cells and medium were collected after the indicated times, specific for each analysis, and analyzed according to the protocols of the different methods.

### 2.3. Toxicity Measurements

Cellular viability was assessed using the 3-(4,5-dimethyl-2-thiazolyl)-2,5-diphenyl-2H-tetrazolium bromide (MTT) assay and the neutral red assay. The MTT assay was performed 4, 24, 48, or 72 hours after Aa treatment by adding 50 *μ*g MTT (Sigma-Aldrich, St. Louis, MO, USA) to each well of a 96-well plate. Plates were protected from light and incubated at +37°C for 90 minutes, after which the MTT medium was removed and the formazan crystals in each well were dissolved in DMSO (Sigma-Aldrich, St. Louis, MO, USA). The optical density of each well was measured at 562 nm with the results being normalized to untreated controls, which were designated as 100% cellular viability. The neutral red assay was performed as previously described by Repetto et al. [25]. Cellular toxicity was determined using the lactate dehydrogenase (LDH) assay (CytoTox 96® Non-Radioactive Cytotoxicity Assay, Promega, Fitchburg, WI, USA). The LDH assay was performed according to the manufacturer's instructions.

### 2.4. Caspase 3 Activity Measurements

Caspase 3 activity was measured from cell lysates collected 24 hours after Aa stimulation. The colorimetric caspase 3 assay (Sigma-Aldrich, St. Louis, MO, USA) was used according to the manufacturer's instructions.

### 2.5. Terminal Deoxynucleotidyl Transferase dUTP Nick End Labeling (TUNEL) Staining

Confluent cells were treated with 25 *μ*M Aa for 24 hours before fixation in 4% paraformaldehyde (PFA) (EMS, Hatfield, PA, USA) and subjected to TUNEL staining. The In Situ Cell Death Detection Fluorescein kit (Roche Diagnostics, Mannheim, Germany) was used according to the manufacturer's instructions for staining cells. Chamber slides were mounted with VECTASHIELD mounting medium containing DAPI (Vector Laboratories, Burlingame, CA, USA), coverslipped, and analyzed using a fluorescence microscope at 20x magnification to assess the number of TUNEL-positive cells. At least 3 images of each well were taken, and the numbers of TUNEL-positive cells, as well as the total number of cells in each image, were counted.

### 2.6. MitoTracker Staining

MitoTracker Green FM (Cell Signaling Technology, Danvers, MA, USA) and MitoTracker Red CMXRos (Thermo Fisher Scientific, Waltham, MA, USA) were used to stain mitochondria in RPE cells. Cell Signaling Technologies was consulted to confirm that their MitoTracker Green FM was sensitive to MMP changes. For assessment of changes to the MMP, fully confluent cells on 96-well plates were exposed to 400 nM MitoTracker Green or Red for 15 minutes after a 4-hour incubation with Aa. Cells were washed with 1x PBS before fluorescence was measured from each well in PBS. The excitation wavelengths were 485 nm and 540 nm while emission wavelengths were 516 nm and 620 nm for MitoTracker Green and MitoTracker Red, respectively. For analysis of MitoTracker Green FM staining using confocal microscopy, cells were split to 8-well chamber slides. Fully confluent cells were treated with Aa for 24 hours, before the medium was removed and cells were exposed first to 400 nM MitoTracker Green FM for 15 minutes, followed by a 10-minute exposure to Hoechst 33342 (Thermo Fisher Scientific, Waltham, MA, USA). Living cells were visualized in fresh medium under a confocal microscope (Zeiss LSM700, Carl Zeiss AG, Oberkochen, Germany) at a magnification of 63x.

### 2.7. Transmission Electron Microscopy (TEM)

In the TEM analyses, cells were grown on 12-well plates (ARPE-19) or on Matrigel-coated glass coverslips (hRPE) until fully confluent. The cells were exposed either to vehicle or to 25 *μ*M Aa for 6 or 24 hours and were then fixed and processed for TEM analysis. All treatment groups and time points were analyzed in duplicate. TEM samples were visualized under a JEM-2100F (JEOL Co., Tokyo, Japan) transmission electron microscope. Images of at least 5 cells from each well were taken at 2000-fold magnification, and the number, shape, and well-being of mitochondria in each cell were assessed. Mitochondrial counting was performed by two independent investigators with the average values of the results being used. Additionally, images were taken at 10000x to generate enlarged images of mitochondrial and autophagosomal structures.

### 2.8. Analysis of Metabolic Potential

An XF^e^96 Extracellular Flux Analyzer (Seahorse Bioscience, North Billerica, MA, USA) was used to analyze the metabolic potential of Aa-treated and untreated RPE cells. Briefly, cells were seeded in a Seahorse 96-well plate and allowed to attach and grow to confluence. The cells were then analyzed for the oxygen consumption rate (OCR) and extracellular acidification rate (ECAR). The instructions of the manufacturer were followed. Four measurements were performed before and five after a single injection with differing concentrations of Aa. After the Seahorse Assay, the cells were lysed in the assay plate and the complete protein amount in each well was measured using the bicinchoninic acid (BCA) assay (Pierce Biotechnology, Rockford, IL, USA) with bovina serum albumin as the standard. The BCA assay was performed according to the manufacturer's instructions, and the protein levels were used to normalize the Seahorse data for each individual well. The results were analyzed using the Seahorse software, and the change in OCR and ECAR before and after Aa injection was calculated to assess the cells' metabolic potential.

### 2.9. Western Blot Analysis

Cells were exposed to vehicle or Aa on 12-well plates before being collected and lysed in M-PER Mammalian Protein Extraction Reagent (Thermo Fisher Scientific, Waltham, MA, USA). The concentration of protein lysates was measured using the Bradford method [26]. Samples were diluted in Milli-Q water to achieve uniform protein concentrations, followed by addition of protein loading dye (Thermo Fisher Scientific, Waltham, MA, USA) and *β*-mercaptoethanol (Sigma-Aldrich, St. Louis, MO, USA) for a final concentration of 5% *β*-mercaptoethanol and 1x loading dye. Western blot samples were separated on a 15% SDS-PAGE gel at 200 V for 3 hours. Protein bands were transferred from the gel to a nitrocellulose membrane (Amersham, Piscataway, NJ, USA) using wet transfer in a blotting buffer containing 20% methanol and incubated overnight at 17 V. Protein transfer to the membranes was confirmed by Ponceau S staining (Sigma-Aldrich, St. Louis, MO, USA). After blocking the membranes in 5% nonfat milk in TBS for 90 minutes, the membranes were probed with primary antibodies overnight at +4°C. The primary antibodies used were anti-LC3 (AP1802a; Abgent, San Diego, CA, USA, 1 : 800), anti-p62 (sc-28359; Santa Cruz Biotechnology, Dallas, TX, USA, 1 : 2000), and anti-tubulin (T5168; Sigma-Aldrich, St. Louis, MO, USA, 1 : 8000). Primary antibody binding was detected using horseradish peroxidase-linked anti-mouse (NA931, GE Healthcare, Chicago, IL, USA, 1 : 25000 for p62 or 1 : 10000 for tubulin) or anti-rabbit (A16104; Invitrogen, Carlsbad, CA, USA, 1 : 10000) antibodies. Secondary antibody bands were detected using the Immobilon™ Western Chemiluminescent HRP Substrate (ECL) (Merck Millipore, Billerica, MA, USA) and visualized on FUJI X-ray films (FUJI Corporation, Tokyo, Japan). Scans of films were analyzed using ImageJ (US National Institutes of Health, Bethesda, MD, USA; http://imagej.nih.gov/ij/).

### 2.10. Transfections

In the transfection experiments, ARPE-19 cells were seeded on 8-well chamber slides at a density of 15000 cells/well and kept at +37°C, 5% CO_2_ overnight, until the cells were 60-80% confluent. Using MEM medium without supplements, dilutions were prepared with Lipofectamine® and of Plus™ Reagent (both Invitrogen by Life Technologies, Carlsbad, CA, USA) with 1.25 *μ*g of an LC3 Tandem plasmid construct. The Tandem plasmid construct was produced and tested in our lab. The Plus™ and plasmid dilutions were added to the Lipofectamine® dilution and incubated for 30 minutes at room temperature. This transfection medium was then added to the cells, which were incubated for 4 hours before the transfection medium was replaced with serum-containing DMEM/F-12 culture medium. After an additional incubation of 20 hours at +37°C and 5% CO_2_, the cells were exposed to vehicle or Aa for 24 hours, before being fixed in 4% PFA. Chamber slides were mounted with DAPI-containing mounting media and coverslips and analyzed under a light microscope (Leica Microsystems, Wetzlar, Germany).

### 2.11. Statistical Analyses

At least three independent repetitions were performed for each experiment, unless otherwise stated. Cell culture experiments were performed in such a way that each condition in an individual experiment was run in parallel in at least three separate, independent wells, unless otherwise stated. Data were combined and analyzed using GraphPad Prism (GraphPad Software Inc., San Diego, CA, USA), and statistical analyses of data points were performed using the Kruskal-Wallis test followed by pairwise comparisons using the Mann-Whitney *U* test. A *P* value of *P* < 0.05 was considered statistically significant.

## 3. Results

### 3.1. Inhibition of Mitochondrial Electron Transport Chain Complex III Induces Cell Death in RPE Cells

**Figure 1:**
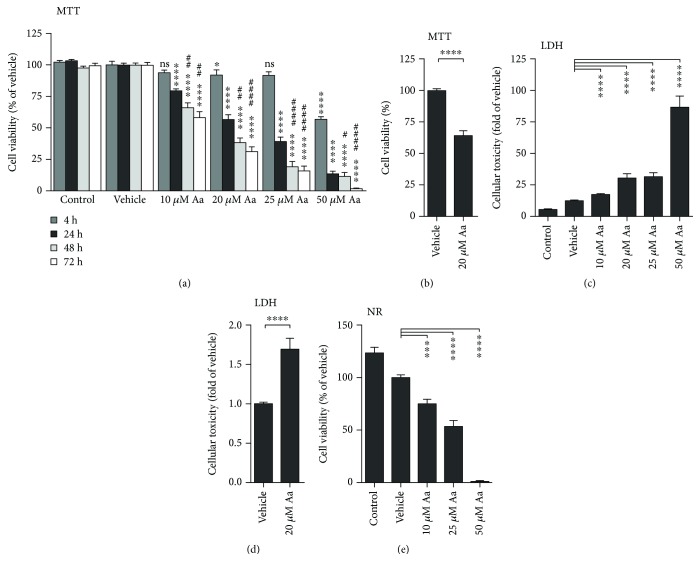
Aa treatment was toxic to human RPE cells. Aa caused a dose- and time-dependent decrease in cellular viability (a, b) and a corresponding increase in cellular toxicity (c) in ARPE-19 cells. In hRPE cells (9 donors), treatment with 20 *μ*M Aa also reduced MTT (d) levels and increased LDH release (e). Experiments were performed at least three times with 4-6 parallels in each group per experiment. Results are presented as mean ± SEM. ns: not statistically significant; ^∗^*P* < 0.05, ^∗∗∗^*P* < 0.001, and ^∗∗∗∗^*P* < 0.0001 compared to vehicle control of the same time point; ^#^*P* < 0.05, ^##^*P* < 0.01, and ^####^*P* < 0.0001 for MTT samples measured 48 or 72 hours after Aa stimulation and compared to the 24-hour time point, Mann-Whitney *U* test.

**Figure 2:**
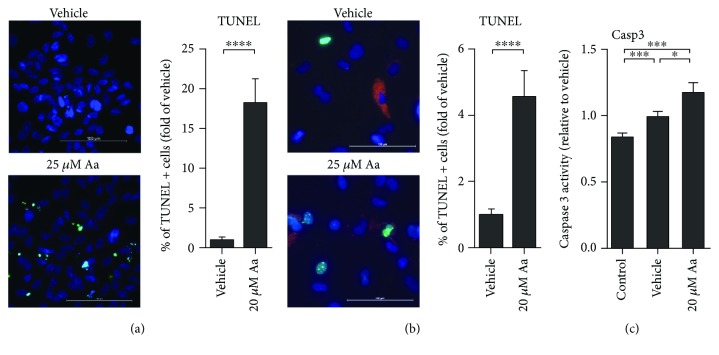
Evidence of apoptosis after Aa exposure. Treatment with Aa led to an increase in the number of TUNEL-positive cells in both ARPE-19 (a) (scale bar: 100 *μ*m; green: TUNEL; blue: DAPI) and hRPE cells (b) (scale bar: 100 *μ*m; green: TUNEL; blue: DAPI; red: autofluorescence of pigment in hRPE cells; 3 different donors). Concurrently, there was an increase in caspase 3 activity in ARPE-19 cells (c). Results are a combination of three independent experiments with 2 (a, b) or 4 (c) parallels per group. Results are presented as mean ± SEM. Microscopy images shown are representative. ^∗^*P* < 0.05 and ^∗∗∗^*P* < 0.001, Mann-Whitney *U* test.

ARPE-19 cells were exposed to increasing concentrations of antimycin A (Aa) to monitor cell death using multiple assays. Aa induced a dose- and time-dependent decrease in cell viability as detected in the MTT assay (Figure 1(a)). ARPE-19 cells also displayed a dose-dependent reduction in neutral red retention (Figure 1(b)) and an increase in LDH leakage (Figure 1(c)). In comparing the response of ARPE-19 with primary cultures of hRPE, all of the cell viability assays returned similar results, though hRPE cells appeared more resistant. In ARPE-19 cells, Aa treatment reduced cell viability to 57% at 20 *μ*M, while hRPE cells retained a viability of 64% (Figures 1(a) and 1(d)). LDH release was 2.4- and 1.7-fold greater than vehicle-treated controls for ARPE-19 and hRPE, respectively (Figures 1(c) and 1(e)). Aa treatment led to a greater number of TUNEL-positive cells in both ARPE-19 and primary human RPE cells (Figures 2(a) and 2(b)) although hRPE appeared slightly more resistant to apoptosis. An increase in caspase 3 activity (Figure 2(c)) in ARPE-19 cells provides further evidence of the involvement of apoptotic pathways.

### 3.2. Antimycin A Induces a Loss of Mitochondrial Membrane Potential

**Figure 3:**
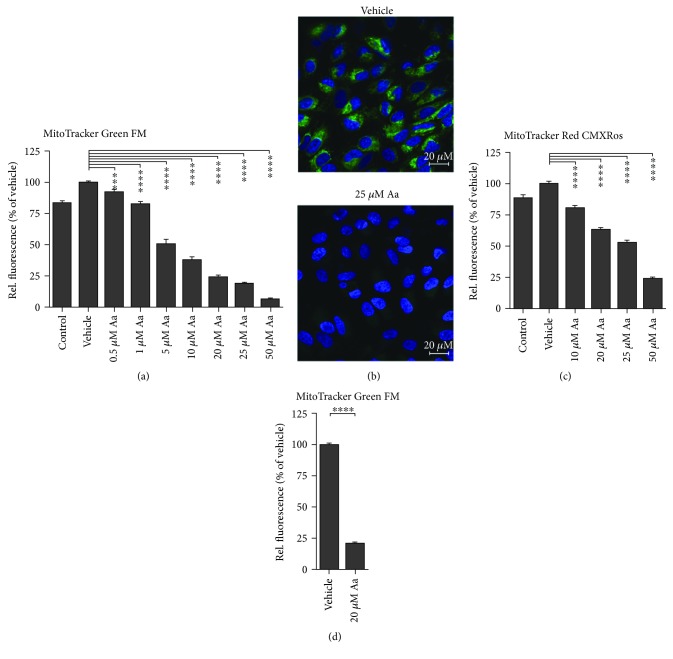
Aa exposure disrupts the MMP. A 4-hour exposure to Aa caused a dose-dependent decrease in MitoTracker Green FM fluorescence in ARPE-19 cells (a). A concentration of 25 *μ*M Aa led to an almost complete loss of MitoTracker fluorescence when observed under the confocal microscope (ARPE-19 cells (b), scale bar: 20 *μ*m; green: MitoTracker Green FM; blue: Hoechst 33342). A second MitoTracker probe, MitoTracker Red CMXRos also showed a dose-dependent reduction in fluorescence activity 4 hours after Aa treatment (c). In hRPE cells treated with 20 *μ*M Aa for 4 hours, MitoTracker Green FM fluorescence was also markedly decreased (9 donors) (d). Results are a combination of six (a), three (c), or ten (d) independent experiments with four (a, c) or 3 (d) parallels per group. Results are presented as mean ± SEM. Microscopy images shown are representative. ^∗∗∗∗^*P* < 0.0001, Mann-Whitney *U* test.

Aa is an inhibitor of complex III of the ETC. Inhibition of the ETC leads to a loss of MMP; this was observed in Aa-treated ARPE-19 and primary hRPE cells (Figure 3). A 4-hour exposure to Aa evoked a dose-dependent decrease in the intensity of the MMP-sensitive stain MitoTracker Green FM in ARPE-19 cells in a 96-well plate-based assay (Figure 3(a)). A clear reduction of MitoTracker fluorescence was also observed under the confocal microscope in ARPE-19 cells treated with 25 *μ*M Aa (Figure 3(b)). Another MMP-sensitive dye, MitoTracker Red CMXRos, confirmed the findings in ARPE-19 cells and showed a similar dose-dependent reduction in fluorescence intensity (Figure 3(c)). In hRPE cells, a 4-hour treatment with 20 *μ*M Aa led to a 78% reduction in the fluorescence intensity of MitoTracker Green FM (Figure 3(d)), which is perfectly in line with the 76% reduction observed in ARPE-19 cells (Figure 3(a)).

### 3.3. Antimycin A Exposure Changes the Phenotype of Mitochondria

**Figure 4:**
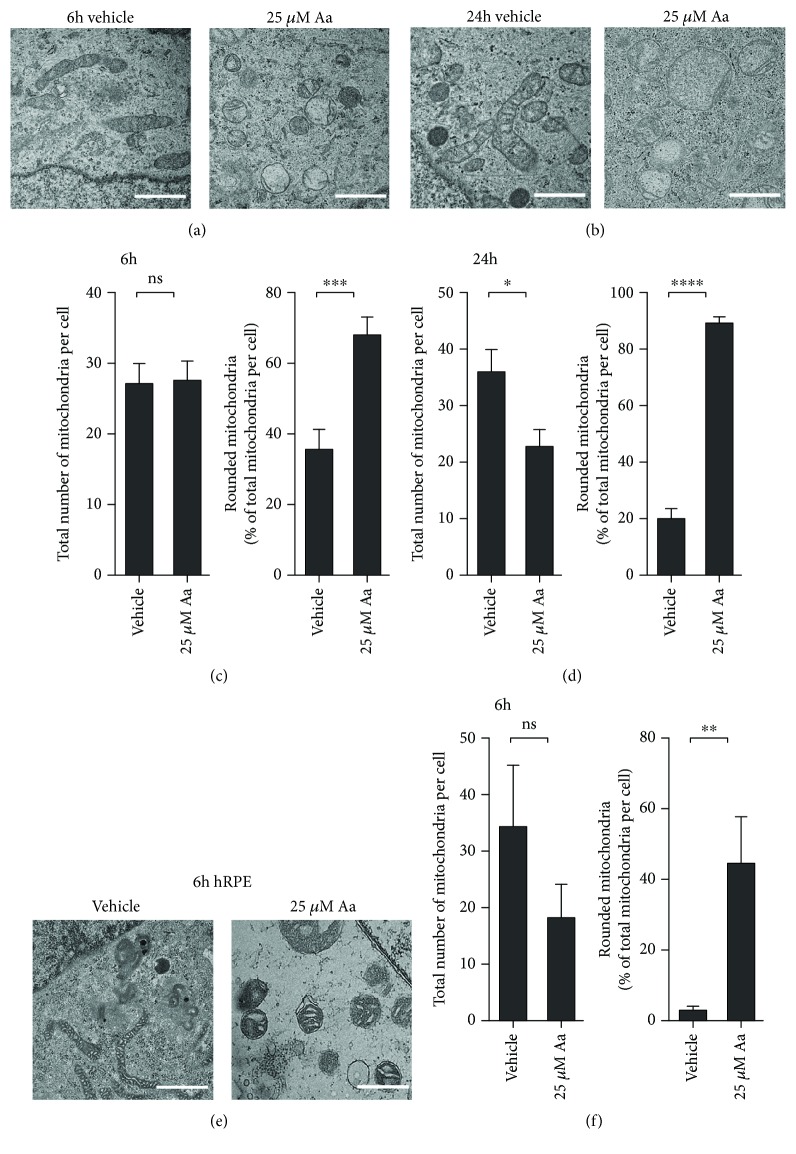
Aa caused significant mitochondrial damage. TEM analysis of the cells revealed mitochondria to be swollen, with clear damage to their cristae and a reduction in the mitochondrial matrix density (a, b, e) (scale bar: 1 *μ*m). At 6 hours, there was no difference in the total number of mitochondria per cell (c), but 24 hours after Aa treatment, the numbers of mitochondria were reduced, while mitochondrial damage persisted (d). Mitochondria in hRPE cells showed similar damage with no reduction in their number after 6 hours of Aa treatment (e, f). Quantitative results are a combination of three (a, b) or two (e) independent experiments, where TEM images of at least 5 separate cells per group were evaluated by two independent researchers. Results are presented as mean ± SEM. Representative TEM images are shown. ns: not statistically significant; ^∗^*P* < 0.05, ^∗∗^*P* < 0.01, ^∗∗∗^*P* < 0.001, and ^∗∗∗∗^*P* < 0.0001, Mann-Whitney *U* test.

Along with the loss of the MMP, mitochondria in Aa-treated RPE cells also underwent drastic changes in their appearance. At 6 hours posttreatment, analysis of TEM images showed no change in the overall number of mitochondria per ARPE-19 cell (Figures 4(a) and 4(c)). However, 68% of the mitochondria were noticeably swollen and showed a loss of cristae and matrix density (Figures 4(a) and 4(c)). At 24 hours post Aa treatment, the percentage of swollen mitochondria made up 89% of the population, and at this later time, the overall number of mitochondria per cell was reduced when compared to those of vehicle-treated ARPE-19 cells (Figures 4(b) and 4(d)). This result suggests that some of the damaged mitochondria had been partially cleared by mitophagy.

Analysis of TEM images from hRPE after a 6-hour incubation with Aa showed no change in total mitochondria, although 44% exhibited a rounded phenotype (Figures 4(e) and 4(f)). Of note, vehicle-treated cells contained rounded mitochondria involving less than 5% of the population in hRPE, whereas nearly 36% of rounded mitochondria were present in the vehicle-treated ARPE-19 cells. These results suggest that the mitochondria in the hRPE were generally healthier and more resistant to Aa-induced mitochondrial damage compared with mitochondria in ARPE-19.

### 3.4. Antimycin A Treatment Triggers a Switch to Glycolytic Pathways in RPE Cells

**Figure 5:**
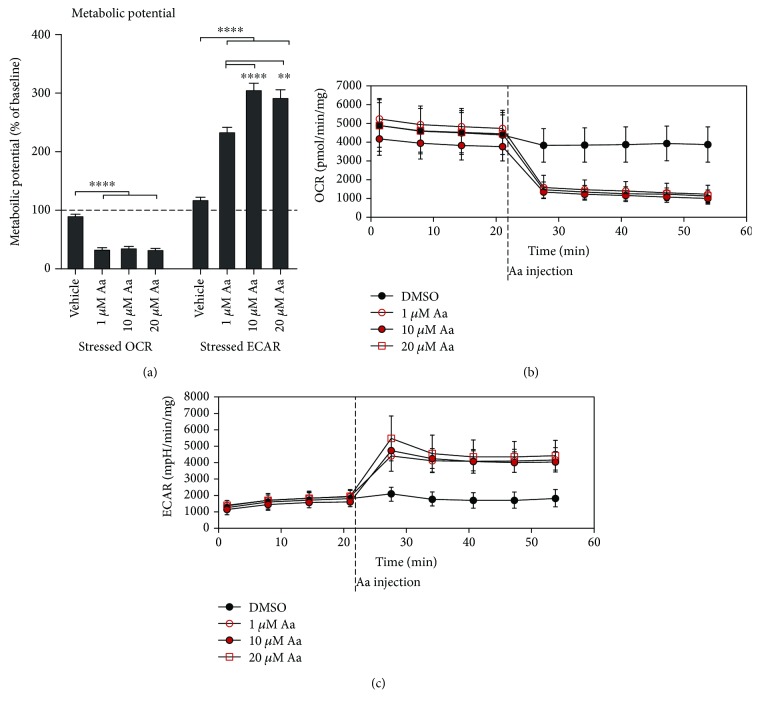
Aa treatment led to the collapse of oxidative phosphorylation in ARPE-19 cells and induced a shift towards glycolysis. Treatment with 1 *μ*M, 10 *μ*M, and 20 *μ*M Aa led to a rapid and major reduction in the oxygen consumption rate (OCR) (a, b) and an increase in the extracellular acidification rate (ECAR) (a, c) in ARPE-19 cells, as measured on an XFe Extracellular Flux Analyzer. Results are combined from three independent experiments with 5 parallels per group. Mean ± SEM is shown. ^∗∗∗∗^*P* < 0.0001, Mann-Whitney *U* test.

As there was both a loss of the MMP and signs of mitochondrial damage, we next analyzed the effect of these changes on mitochondrial function. We utilized a Seahorse Analyzer to measure both the oxygen consumption rate (OCR), an index of mitochondrial respiration, and the extracellular acidification rate (ECAR), a measure of glycolysis. Not surprisingly, Aa treatment caused a drastic and fast reduction in mitochondrial respiration, confirming the loss of mitochondrial function (Figures 5(a) and 5(b)). Without the energy production by OXPHOS and in order to facilitate cellular survival, ARPE-19 cells had activated glycolysis pathways, as evidenced by an increase in ECAR (Figures 5(a) and 5(c)).

### 3.5. RPE Cells Employ Mitophagy to Limit Cell Death after Mitochondrial Damage

**Figure 6:**
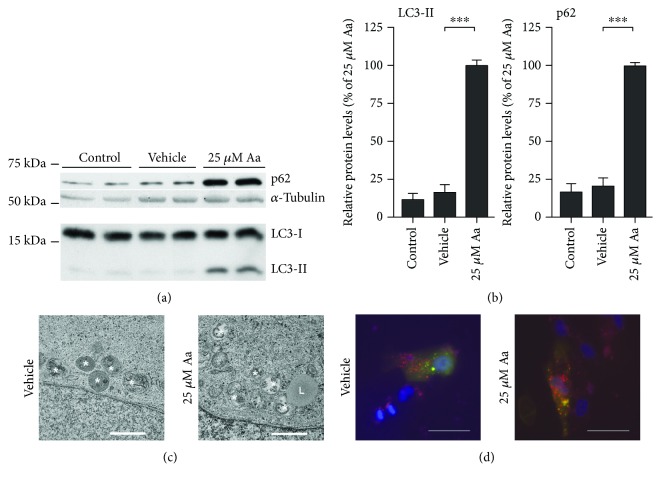
Autophagy was active in Aa-treated ARPE-19 cells. Treatment with 25 *μ*M Aa for 24 hours led to a marked increase in the protein levels of p62 and LC3-II (a, b). Autophagic vesicles were visible also in the TEM analysis (c) (white asterisks: autophagic vesicles; L: lysosome; scale bar: 1 *μ*m), and cells transfected with a LC3 Tandem plasmid construct showed clear punctae indicating either autophagophores (yellow) or autophagosomes (red) (d) (scale bar: 20 *μ*m; blue: DAPI). Western blot data is combined from three independent experiments with four parallels per group. Cropped regions of representative blots and representative microscopy images are shown. Quantitative results are presented as mean ± SEM. ^∗∗∗^*P* < 0.001, Mann-Whitney *U* test.

**Figure 7:**
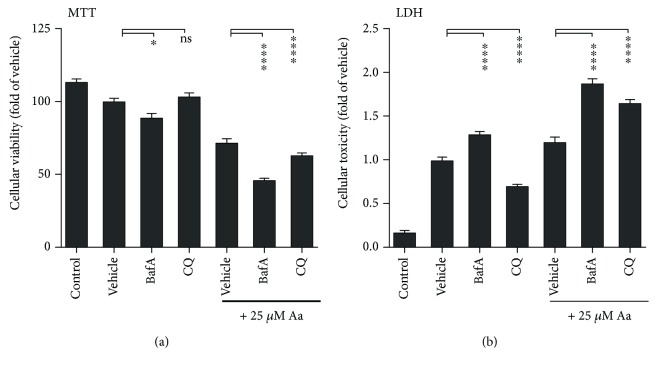
Inhibition of autophagy increased cell death in antimycin A- (Aa-) treated ARPE-19 cells. Inhibition of autophagy in Aa-treated cells using 50 nM bafilomycin A1 (BafA) or 30 *μ*M chloroquine (CQ) decreased cellular viability measured by the MTT assay and increased the release of LDH into the culture medium. This increased cell toxicity was greater after Aa treatment than in the absence of mitochondrial damage. Results are combined from three independent experiments with six parallels per group. Results are presented as mean ± SEM. ns: not statistically significant; ^∗^*P* < 0.05 and ^∗∗∗∗^*P* < 0.0001, Mann-Whitney *U* test.

The decrease in the total number of mitochondria per cell 24 hours after Aa treatment suggests that the cells had initiated the clearance of damaged mitochondria. We observed an increase in the levels of the autophagy-related proteins, Sequestosome1/p62 (p62) and the lipidated form of the microtubule-associated protein 1 light chain 3 (LC3), and LC3-II, 24 hours after the addition of Aa, suggesting the upregulation of autophagy (Figures 6(a) and 6(b)). In support of this, both TEM and fluorescence microscopy analysis of ARPE-19 cells transfected with recombinant LC3 revealed the presence of autophagic structures in RPE cells (Figures 6(c) and 6(d)). Finally, we inhibited macroautophagy, using either bafilomycin A1 (BafA) or chloroquine (CQ) in Aa-treated RPE cells, and found that autophagy inhibition augmented cell death (Figure 7). This further supports the hypothesis that Aa-treated cells were utilizing mitophagy to clear the damaged mitochondria and limit cellular toxicity.

## 4. Discussion

AMD is the leading cause of blindness among the elderly in the Western World [12]. This disease affects the RPE, a single-cell layer responsible for the upkeep and protection of the overlying photoreceptor cells. The RPE is a postmitotic tissue that has been shown to accumulate mtDNA mutations [14, 15] and mitochondrial damage with age [13]. Karunadharma and colleagues also reported an age-dependent increase in the “common deletion,” a region of mtDNA that is particularly susceptible to deletion in postmitotic cells [27]. Consequently, it has been suggested that mitochondrial damage may be a key player in RPE cell death and the development of AMD [13, 15, 27]. Until now, most research has focused on investigations using retinal tissue collected posthumously from aged donors or AMD patients [13–16, 28].

In our new cell-based model, treatment of human RPE cells with the complex III inhibitor Aa led to a robust dose- and time-dependent decrease in cell viability that was preceded by a rapid and significant loss of the mitochondrial membrane potential (MMP), mitochondrial swelling, and a failure of OXPHOS. Previous reports have described Aa treatment to evoke a similar increase in cell death preceded by a loss of MMP in endothelial cells [23], osteoblasts [29], pulmonary fibroblasts [30], and hepatocytes [22]. Many of the studied cells also displayed an increase in caspase activity and a leakage of proapoptotic factors from the mitochondria, similar to what we observed in RPE cells [22, 23, 30].

Of particular note is that the results that we obtained in the immortalized cell line ARPE-19 were generally reproducible in primary human RPE cells. While hRPE cells showed a slightly stronger resistance to the cytotoxic and mitochondria-damaging effects of Aa treatment, they also suffered from a loss of MMP, mitochondrial swelling, and considerable cell death. Primary hRPE cells generated from aged donors are a valuable model that can recapitulate many features of RPE from aged patients in an *in vitro* setting. However, the high cost and the limited availability of donor samples make this model unattainable for most laboratories. Our findings that hRPE cells and ARPE-19 cells react in a similar manner to Aa exposure not only increases the clinical relevance of our findings but also suggests that ARPE-19 cells are an important first model prior to subsequent confirmatory studies in more complex models, such as patient-derived RPE cells, or rodent models expressing the disease phenotype.

Concurrently with the increased cell death, we observed an increase in intracellular LC3-II levels, possibly suggesting an increase in autophagophore formation; TEM and transfection studies verified the presence of autophagosomal vesicles inside RPE cells. Additionally, there was an increase in the levels of p62, an adapter protein that can facilitate the link of poly-ubiquitinated mitochondria, i.e., mitochondria marked for mitophagy, with the autophagophore [31]. This is in line with previous reports by Zhou et al., who described an increase in autophagophore formation close to mitochondria after the inhibition of complex I of the ETC in macrophages [32], and Lee et al., who reported an increase in autophagy in RPE cells treated with rotenone [20]. Both groups suggested that mitophagy was an important process in reducing mitochondrial damage-related cellular toxicity. Autophagy activation may be linked to the generation of superoxide, which has been shown to be the key ROS regulator of autophagy [17]. Like complex I, complex III is a major source of mitochondrial superoxide [17] and Aa has been shown to increase the amounts of intracellular ROS in hepatocytes [22], osteoblasts [29], and neurons [33]. Further studies on the importance of mitochondrial ROS after Aa treatment in RPE cells may shed light on the pathway linking mitochondrial damage to autophagy.

In line with the results reported by Lee et al. after rotenone treatment [20], in our model, we found that inhibition of autophagy provoked increased cell death. This suggests that cells actively employ autophagy/mitophagy as a protective mechanism to limit mitochondria-related cell damage. It is interesting to consider that with advanced age and in AMD patients, the autophagic capabilities of RPE cells decline, leading to the accumulation of protein aggregates and possibly damaged mitochondria [18, 34, 35]. It would be interesting to investigate whether activation of mitophagy or a decrease in the levels of mitochondrial ROS could reduce cell death in RPE cells suffering from mitochondrial damage.

Finally, Zhou et al. showed that inhibition of complex III using Aa or complex I using rotenone led to increased ROS production in THP1 macrophages which triggered activation of inflammasomes [32]. They suggested that this represents an additional role for mitochondria—regulation of inflammatory reactions. It is well established that chronic inflammation plays a decisive role in RPE cell death and AMD development. By unravelling the mechanisms behind mitochondrial damage-related inflammation in RPE cells, it may be possible to clarify the role of mitochondria in disease biogenesis.

## 5. Conclusions

In summary, RPE cells are believed to accumulate damaged mitochondria with age as damaged mtDNA levels increase and clearing mechanisms fail. This proposal is in line with the report of Feher et al. that the numbers of mitochondria, the integrity of their cristae structure, and mitochondrial matrix density all decrease with age and that this process occurs prematurely in AMD patients [13]. In our cells, Aa treatment successfully duplicated the damage to mitochondria seen in patient tissue samples and led to dose-dependent cell death. Autophagy remained active despite damage to mitochondria and was involved in minimizing the Aa-dependent cell death. These findings indicate that Aa treatment might be a suitable model for investigating the effects of mitochondrial damage in RPE cells and highlight the importance of functional mitophagy.

## Figures and Tables

**Table 1:** Donor characteristics and passage number of human primary RPE cells used in this study.

Number of donors (*n*)	Sex		Age	Passage number of hRPE cells (*n*)
	Male	Female	(mean ± SD)	
9	6	3	71.4 ± 7.6	P3 (7), P4 (2)

## Data Availability

The data used to support the findings of this study are included within the article.

## References

[1] Greaves L. C., Reeve A. K., Taylor R. W., Turnbull D. M. (2012). Mitochondrial DNA and disease. *The Journal of Pathology*.

[2] Vakifahmetoglu-Norberg H., Kim M., Xia H. G. (2013). Chaperone-mediated autophagy degrades mutant p53. *Genes & Development*.

[3] Kauppinen A., Fulop T., Franceschi C., Hirokawa K., Pawelec G. (2018). Mitochondria-associated inflammasome activation and its impact on aging and age-related diseases. *Handbook on Immunosenescence*.

[4] Jang Y. C., Van Remmen H. (2009). The mitochondrial theory of aging: insight from transgenic and knockout mouse models. *Experimental Gerontology*.

[5] Pinto M., Moraes C. T. (2014). Mitochondrial genome changes and neurodegenerative diseases. *Biochimica et Biophysica Acta (BBA) - Molecular Basis of Disease*.

[6] Jeppesen D. K., Bohr V. A., Stevnsner T. (2011). DNA repair deficiency in neurodegeneration. *Progress in Neurobiology*.

[7] Fukae J., Mizuno Y., Hattori N. (2007). Mitochondrial dysfunction in Parkinson’s disease. *Mitochondrion*.

[8] Bender A., Krishnan K. J., Morris C. M. (2006). High levels of mitochondrial DNA deletions in substantia nigra neurons in aging and Parkinson disease. *Nature Genetics*.

[9] Chen J. X., Yan S. S. (2010). Role of mitochondrial amyloid-*β* in Alzheimer’s disease. *Journal of Alzheimer's Disease*.

[10] Sheng B., Wang X., Su B. (2012). Impaired mitochondrial biogenesis contributes to mitochondrial dysfunction in Alzheimer’s disease. *Journal of Neurochemistry*.

[11] Kauppinen A., Paterno J. J., Blasiak J., Salminen A., Kaarniranta K. (2016). Inflammation and its role in age-related macular degeneration. *Cellular and Molecular Life Sciences*.

[12] Tomany S. C., Wang J. J., Van Leeuwen R. (2004). Risk factors for incident age-related macular degeneration: pooled findings from 3 continents. *Ophthalmology*.

[13] Feher J., Kovacs I., Artico M., Cavallotti C., Papale A., Balacco Gabrieli C. (2006). Mitochondrial alterations of retinal pigment epithelium in age-related macular degeneration. *Neurobiology of Aging*.

[14] Kenney M. C., Atilano S. R., Boyer D. (2010). Characterization of retinal and blood mitochondrial DNA from age-related macular degeneration patients. *Investigative Ophthalmology & Visual Science*.

[15] Terluk M. R., Kapphahn R. J., Soukup L. M. (2015). Investigating mitochondria as a target for treating age-related macular degeneration. *The Journal of Neuroscience*.

[16] Nordgaard C. L., Karunadharma P. P., Feng X., Olsen T. W., Ferrington D. A. (2008). Mitochondrial proteomics of the retinal pigment epithelium at progressive stages of age-related macular degeneration. *Investigative Ophthalmology & Visual Science*.

[17] Chen Y., Azad M. B., Gibson S. B. (2009). Superoxide is the major reactive oxygen species regulating autophagy. *Cell Death and Differentiation*.

[18] Hyttinen J. M. T., Blasiak J., Niittykoski M. (2017). DNA damage response and autophagy in the degeneration of retinal pigment epithelial cells—implications for age-related macular degeneration (AMD). *Ageing Research Reviews*.

[19] Srivastava S. (2017). The mitochondrial basis of aging and age-related disorders. *Genes*.

[20] Lee S. Y., Oh J. S., Rho J. H. (2014). Retinal pigment epithelial cells undergoing mitotic catastrophe are vulnerable to autophagy inhibition. *Cell Death & Disease*.

[21] Hyttinen J. M. T., Viiri J., Kaarniranta K., Blasiak J. (2018). Mitochondrial quality control in AMD: does mitophagy play a pivotal role?. *Cellular and Molecular Life Sciences*.

[22] Guha G., Mandal T., Rajkumar V., Ashok Kumar R. (2010). Antimycin A-induced mitochondrial apoptotic cascade is mitigated by phenolic constituents of Phyllanthus amarus aqueous extract in Hep3B cells. *Food and Chemical Toxicology*.

[23] You B. R., Park W. H. (2010). The effects of antimycin A on endothelial cells in cell death, reactive oxygen species and GSH levels. *Toxicology In Vitro*.

[24] Ferrington D. A., Ebeling M. C., Kapphahn R. J. (2017). Altered bioenergetics and enhanced resistance to oxidative stress in human retinal pigment epithelial cells from donors with age-related macular degeneration. *Redox Biology*.

[25] Repetto G., del Peso A., Zurita J. L. (2008). Neutral red uptake assay for the estimation of cell viability/cytotoxicity. *Nature Protocols*.

[26] Bradford M. M. (1976). A rapid and sensitive method for the quantitation of microgram quantities of protein utilizing the principle of protein-dye binding. *Analytical Biochemistry*.

[27] Karunadharma P. P., Nordgaard C. L., Olsen T. W., Ferrington D. A. (2010). Mitochondrial DNA damage as a potential mechanism for age-related macular degeneration. *Investigative Ophthalmology & Visual Science*.

[28] Rohrer B., Bandyopadhyay M., Beeson C. (2016). Reduced metabolic capacity in aged primary retinal pigment epithelium (RPE) is correlated with increased susceptibility to oxidative stress. *Advances in Experimental Medicine and Biology*.

[29] Choi E. M., Lee Y. S. (2011). Mitochondrial defects and cytotoxicity by antimycin A on cultured osteoblastic MC3T3-E1 cells. *Food and Chemical Toxicology*.

[30] Park W. H., You B. R. (2016). Antimycin A induces death of the human pulmonary fibroblast cells via ROS increase and GSH depletion. *International Journal of Oncology*.

[31] Yamaguchi O., Murakawa T., Nishida K., Otsu K. (2016). Receptor-mediated mitophagy. *Journal of Molecular and Cellular Cardiology*.

[32] Zhou R., Yazdi A. S., Menu P., Tschopp J. (2011). A role for mitochondria in NLRP3 inflammasome activation. *Nature*.

[33] Leuner K., Schutt T., Kurz C. (2012). Mitochondrion-derived reactive oxygen species lead to enhanced amyloid beta formation. *Antioxidants & Redox Signaling*.

[34] Kaarniranta K., Sinha D., Blasiak J. (2013). Autophagy and heterophagy dysregulation leads to retinal pigment epithelium dysfunction and development of age-related macular degeneration. *Autophagy*.

[35] Golestaneh N., Chu Y., Xiao Y. Y., Stoleru G. L., Theos A. C. (2017). Dysfunctional autophagy in RPE, a contributing factor in age-related macular degeneration. *Cell Death & Disease*.

